# Green finance growth prediction model based on time-series conditional generative adversarial networks

**DOI:** 10.1371/journal.pone.0306874

**Published:** 2024-07-24

**Authors:** Aya Salama Abdelhady, Nadia Dahmani, Lobna M. AbouEl-Magd, Ashraf Darwish, Aboul Ella Hassanien

**Affiliations:** 1 Faculty of Mathematical and Computational Sciences, University of Prince Edward Island, Charlottetown, Canada; 2 Scientific Research School of Egypt (SRSEG), Cairo, Egypt; 3 College of Technological Innovation, Zayed University, Dubai, UAE; 4 LARODEC, Institut Supérieur de Gestion de Tunis, Université de Tunis, Tunis, Tunisia; 5 Computer Science Department, Misr Higher Institute, Mansoura, Egypt; 6 Faculty of Science, Helwan University, Helwan, Egypt; 7 Egyptian Chinese University, Cairo, Egypt; 8 Faculty of Computers and AI, Cairo University, Cairo, Egypt; 9 College of Business Administration (CBA), Kuwait University, Kuwait City, Kuwait; UCSI University Kuala Lumpur Campus: UCSI University, MALAYSIA

## Abstract

Climate change mitigation necessitates increased investment in green sectors. This study proposes a methodology to predict green finance growth across various countries, aiming to encourage such investments. Our approach leverages time-series Conditional Generative Adversarial Networks (CT-GANs) for data augmentation and Nonlinear Autoregressive Neural Networks (NARNNs) for prediction. The green finance growth predicting model was applied to datasets collected from forty countries across five continents. The Augmented Dickey-Fuller (ADF) test confirmed the non-stationary nature of the data, supporting the use of Nonlinear Autoregressive Neural Networks (NARNNs). CT-GANs were then employed to augment the data for improved prediction accuracy. Results demonstrate the effectiveness of the proposed model. NARNNs trained with CT-GAN augmented data achieved superior performance across all regions, with R-squared (R^2^) values of 98.8%, 96.6%, and 99% for Europe, Asia, and other countries respectively. While the RMSE for Europe, Asia, and other countries are 1.26e+2, 2.16e+2, and 1.16e+2 respectively. Compared to a baseline NARNN model without augmentation, CT-GAN augmentation significantly improved both R^2^ and RMSE. The R^2^ values for the Europe, Asia, and other countries models are 96%, 73%, and 97.2%, respectively. The RMSE values for the Europe, Asia, and various countries models are 2.24e+2, 7e+2, and 2.07e+2, respectively. The Nonlinear Autoregressive Exogenous Neural Network (NARX-NN) exhibited significantly lower performance across Europe, Asia, and other countries with R2 values of 74%, 52%, and 86%, and RMSE values of 1.11e+2, 3.63e+2, and 1.8e+2, respectively.

## Introduction

Global warming and climate change are considered as the biggest economic failures and challenging situations. Earth’s atmosphere is witnessing a huge concentration of carbon dioxide, almost more than 420 parts per million (ppm) as per NASA’s data [1]. Accordingly, tackling the challenges of global warming and reducing air pollution falls not only on these nations but is a collective responsibility shared by all of humanity [2, 3].

Since the 1960s, national and international policymakers, economists, and environmental activists have been more conscious of the damaging effects of environmental degradation on climate change. Subsequently, to promote economic development, numerous nations have put forth laws and policies to combat environmental deterioration. To guarantee a clean, safe, healthy, and productive environment, for example, Malaysia implemented the Environmental Quality Act in 1974 [4]. Increasing economic growth is linked to increasing levels of environmental pollution to increase growth engines that depend on consumer and manufacturing activities to meet societal requirements, which in turn causes wasteful pollution and strains environmental resources [4].

There are some commitments made by many countries, including China, against climate change, and some of these pledges include developing the renewable energy industry and modernizing the energy system. Policymakers and authorities have made extensive efforts to make this a reality [5].

Accordingly, the need for green financing has developed to achieve long-term growth and sustainable development, as green financing is defined as financial investments aimed at sustainable development projects that protect the environment. Green finance has many types such as climate finance, industrial pollution control, water sanitation, and biodiversity protection. The main goal of green finance is to protect the environment by reducing or avoiding emissions of greenhouse gases (GHGs).

For all the above, green finance is one of the most important areas of research. This concept has been widely addressed in Western countries that have had the greatest impact on the environment, such as China [6].

While artificial intelligence (AI) facilitates greater efficiency in marketing, creativity has been emphasized as the future of business. Existing theories and frameworks in the literature have failed to adequately explore the impact of AI on investment innovation [7].

This study aims to introduce a model for forecasting the expansion of green finance using time-series conditional generative adversarial networks. The proposed model employs artificial intelligence algorithms on publicly available data to construct a green finance recommendation system capable of forecasting the overall volume of green investments globally. Below are the principal contributions of this research:

- Forecasting the growth of green finance worldwide were proposed
- Incorporating CT-GAN to address data scarcity issues.
- Utilizing a straightforward neural network (NAR-NN) suitable for the dataset’s characteristics.

## Related work

According to the studied literature, there are few research investigations that measure the impact of pollution on investments and capital flow [8]. In these studies, researchers have usually relied on mathematical tools such as stochastic calculus [9], random processes, ARIMA time series regression [10], and GARCH volatility models [11] to detect the various time-series patterns. However, the value of financial assets is influenced by an array of factors spanning both financial and non-financial domains. Accordingly, this complexity renders traditional models inadequate.

Authors in [12], discussed the importance of analyzing and forecasting carbon emissions, energy consumption, and the outputs for transitioning to a clean energy economy, especially in rapidly growing markets like China. The paper utilized a nonlinear grey Bernoulli model (NGBM) to predict these indicators and proposed a method to optimize its parameters. The results indicated that the forecasting ability of NGBM with optimized parameters (NGBM-OP) outperforms traditional models like GM and ARIMA, with Mean Absolute Percentage Errors (MAPEs) ranging from 1.10 to 6.26 for out-of-sample data (2004–2009).

The predictions also suggested that between 2011 and 2020, China’s compound annual emissions are expected to grow by 4.47%, while energy consumption was forecasted to decrease slightly (-0.06%), and real GDP is expected to increase by 6.67%. Moreover, authors in [5] highlighted the strategic importance of developing renewable energy. Through a time-series analysis, this research revealed that financial development contributed significantly, explaining 42.42% of the variation in renewable energy growth. Capital market development emerges as the most crucial factor, followed by foreign investment. A comparison with the EU and the US cases suggested that the EU’s approach is more relevant and warrants careful study by Chinese policymakers.

Furthermore, in [13], authors were developing the renewable energy sector and upgrading China’s energy structure play pivotal roles in addressing climate change commitments. Financial issues emerge as a critical constraint, directly tied to the country’s financial development. The study proved that financial development contributes significantly, with capital market development being the most crucial factor, followed by foreign investment, advocating for a closer examination of the EU’s approach by Chinese policymakers.

Three significant contributions have been presented in [14]. First, the authors started by talking about the evolution of the financial well-being domain. Second, they put forth a theoretical framework that delineates the antecedents-based interventions that can be implemented in a particular socioeconomic context to achieve economic well-being. Third, a list of methodological and topical propositions was provided for future researchers and academics to review. They also developed ten future research agendas (FRAs) concerning financial well-being, addressing the need to examine diverse nations with diverse market structures.

On the other side, Machine learning techniques enable investors to enhance financial assets prediction and forecast market strength more accurately than conventional methods. The advent of advanced computer technology such as deep neural networks, and Long Short-Term Memory (LSTM) networks, have prompted a shift toward capturing complex information impacting financial assets [15]. LSTM networks excel at retaining long-term information, unlike traditional models. In addition, Convolutional Neural Networks (CNNs), were adopted to extract features and recognize local dependencies [16].

Combining CNN and LSTM, a model known as ConvLSTM2D was proposed in [17], this research proposed a regression and neural network technique to model stock prices alongside environmental factors, aiming to offer a more precise time series model for stock prices. The model incorporated the ConvLSTM2D network, which extracted all necessary information from air pollution data from major industrialized Chinese cities including Beijing, Taiyuan, Changchun, and Shijiazhuang. Furthermore, Bidirectional LSTM was used in [18] to in investigate how air pollutants indirectly influence investor sentiment and endeavors to establish a more comprehensive and effective stock price prediction framework. The study focused on the SSE Shanghai Enterprises (SSESHE) index and introduced six distinct air pollutants as crucial input parameters. The predictive model developed both, Bidirectional and Long Short-Term Memory (BiLSTM) to project stock closing prices. Additionally, the study compared the proposed model against Support Vector Regression (SVR), Long Short-Term Memory (LSTM), and Gate Recurrent Unit (GRU) models. The experiments concluded that the BiLSTM model that integrated air pollutant data in stock forecasting, achieved the highest prediction accuracy of 94.1%.

According to the conducted literature review conducted, the impact of pollution on investments in green finance specifically has never been addressed, despite its importance in measuring the evolution of green finance over the years. Consequently, this research focuses on studying neural time series techniques that can evaluate the success of green finance across various time periods. To aid in analysis and forecasting issues within the tested dataset of investments in green finance across continents over the years, the nonlinear autoregressive neural network (NAR-NN) and NAR-NN have been explored.

## Economic growth of the studied countries

In this paper, we analyze green finance from 40 different countries across 5 continents. Table 1 summarizes the financial status of these countries. The Gross Domestic Product (GDP) represents the total monetary value of all goods and services produced and sold within a country for one year. The global GDP is estimated to be $100,562,000,000,000. Among the countries studied, Tunisia stood out as the sole representative of Africa. Classified as an upper-middle-income country, Tunisia’s Gross Domestic Product (GDP) grew at an annual rate of 3.5% in the pre-revolution period, from 2008 to 2010 [19]. A research program outlined in [20] proposes a multilevel and multidisciplinary approach to financial system policy, aiming for environmental, social, and economic sustainability. The program leverages social sciences to teach students how financial tools can address economic, social, and environmental challenges. A key focus is on achieving the European Union’s "Europe 2030" goals, which require an estimated annual investment of EUR 180 billion for the next 20 years, particularly in Central and Eastern Europe, to improve energy efficiency and reduce transport emissions. According to [21], the green bond market, a specific segment focused on climate-friendly projects, was launched in 2007–2008 with the help of the first offerings from Multilateral Development Banks. This market has seen a surge in participation from sub-national agencies, local development funds, and institutions like the World Bank, International Monetary Fund, and the European Investment Bank, particularly between 2007 and 2012.

**Table 1:** Financial analysis of the studied countries.

Index	Country	GDP (nominal, 2022)	GDP (Abbrev.)	GDP growth	Population (2022)	GDP per capita
1	Brunei	$16, 681, 531, 646	$16.68 billion	−1.63%	449,002	$37, 152
2	Malta	$17, 765, 270, 015	$17.77 billion	6.85%	533,286	$33, 313
3	Iceland	$27, 841, 648, 044	$27.84 billion	6.44%	372,899	$74, 663
4	Cyprus	$28, 439, 052, 741	$28.44 billion	5.63%	1, 251, 488	$22, 724
5	Estonia	$38, 100, 812, 959	$38.10 billion	−1.29%	1, 326, 062	$28, 732
6	Latvia	$41, 153, 912, 663	$41.15 billion	1.98%	1, 850, 651	$22, 238
7	Tunisia	$46, 664, 948, 952	$46.66 billion	2.52%	12, 356, 117	$3, 777
8	Slovenia	$62, 117, 768, 015	$62.12 billion	5.37%	2, 119, 844	$29, 303
9	Costa Rica	$68, 380, 838, 316	$68.38 billion	4.31%	5, 180, 829	$13, 199
10	Lithuania	$70, 334, 299, 008	$70.33 billion	**1**.**89**%	2, 750, 055	$25, 576
11	Croatia	$70, 964, 606, 465	$70.96 billion	6.33%	4, 030, 358	$17, 608
12	Bulgaria	$89, 040, 398, 406	$89.04 billion	3.36%	6, 781, 953	$13, 129
13	Slovakia	$115, 469, 000, 000	$115 billion	1.67%	5, 643, 453	$20, 461
14	Greece	$219, 066, 000, 000	$219 billion	5.91%	10, 384, 971	$21, 095
15	Kazakhstan	$220, 623, 000, 000	$221 billion	3.20%	19, 397, 998	$11, 373
16	Peru	$242, 632, 000, 000	$243 billion	2.68%	34, 049, 588	$7, 126
17	Portugal	$251, 945, 000, 000	$252 billion	6.69%	10, 270, 865	$24, 530
18	Finland	$280, 826, 000, 000	$281 billion	2.08%	5, 540, 745	$50, 684
19	Czech Republic	$290, 924, 000, 000	$291 billion	2.46%	10, 493, 986	$27, 723
20	Colombia	$343, 939, 000, 000	$344 billion	7.50%	51, 874, 024	$6, 630
21	Hong Kong	$359, 839, 000, 000	$360 billion	−3.48%	7, 488, 865	$48, 050
22	Philippines	$404, 284, 000, 000	$404 billion	7.57%	115, 559, 009	$3, 499
23	Malaysia	$406, 306, 000, 000	$406 billion	8.69%	33, 938, 221	$11, 972
24	Ireland	$529, 245, 000, 000	$529 billion	11.97%	5, 023, 109	$105, 362
25	Belgium	$578, 604, 000, 000	$579 billion	3.25%	11, 655, 930	$49, 640
26	Norway	$579, 267, 000, 000	$579 billion	3.28%	5, 434, 319	$106, 594
27	Argentina	$632, 770, 000, 000	$633 billion	5.24%	45, 510, 318	$13, 904
28	Switzerland	$807, 706, 000, 000	$808 billion	2.06%	8, 740, 472	$92, 410
29	Turkey	$905, 988, 000, 000	$906 billion	**5**.**57**%	85, 341, 241	$10, 616
30	Netherlands	$991, 115, 000, 000	$991 billion	4.48%	17, 564, 014	$56, 429
31	Indonesia	$1, 319, 100, 000, 000	$1.319 trillion	5.31%	275, 501, 339	$4, 788
32	Denmark	$395, 404, 000, 000	$395 billion	3.82%	5, 882, 261	$67, 220
33	Spain	$1, 397, 510, 000, 000	$1.398 trillion	5.45%	47, 558, 630	$29, 385
34	South Korea	$1, 665, 250, 000, 000	$1.665 trillion	2.56%	51, 815, 810	$32, 138
35	Brazil	$1, 920, 100, 000, 000	$1.920 trillion	2.90%	215, 313, 498	$8, 918
36	Italy	$2, 010, 430, 000, 000	$2.010 trillion	3.67%	59, 037, 474	$34, 053
37	Canada	$2, 139, 840, 000, 000	$2.140 trillion	3.40%	38, 454, 327	$55, 646
38	Russia	$2, 240, 420, 000, 000	$2.240 trillion	−2.07%	144, 713, 314	$15, 482
39	France	$2, 782, 910, 000, 000	$2.783 trillion	2.56%	64, 626, 628	$43, 061
40	Germany	$4, 072, 190, 000, 000	$4.072 trillion	1.79%	83, 369, 843	$48, 845
41	Japan	$4, 231, 140, 000, 000	$4.231 trillion	1.03%	123, 951, 692	$34, 135
42	China	$17, 963, 200, 000, 000	$17.963 trillion	2.99%	1, 425, 887, 337	$12, 598

In Europe, Turkey stands out as an upper-middle-income country with a mixed-market emerging economy, reflecting its ongoing economic development and growth [22]. Shifting to North America, Costa Rica, a Central American nation, is another upper-middle-income country that has witnessed steady economic expansion over the past 25 years [23]. Canada, also in North America, boasts the world’s ninth-largest economy and maintains strong trade partnerships with the United States, China, and the United Kingdom [24]. Finally, in Asia, Japan reigns supreme as the world’s third-largest economy. Moreover, Japan’s position as the world’s leading creditor nation grants it significant global influence with far-reaching economic implications [25].

## Preliminaries

### GAN for data augmentation

Machine learning algorithms often struggle with imbalanced datasets, where one class has significantly more samples than others. To address this challenge, we can leverage two techniques: Generative Adversarial Networks (GANs) and Synthetic Minority Over-sampling Technique (SMOTE). While SMOTE is a useful tool, it can create new samples too similar to the majority class, leading to overfitting and poor model performance. In contrast, GANs excel at learning the distribution of the minority class, generating more representative samples. Additionally, GANs offer a robust way to enrich existing data. These networks consist of two key components: a generator and a discriminator. The generator synthesizes new data points, while the discriminator attempts to distinguish real data from the generated samples. Through this adversarial process, the generator learns to create increasingly realistic synthetic data that fools the discriminator [26].

Generative Adversarial Networks (GANs) offer an alternative to conventional augmentation techniques by generating synthetic samples resembling the minority class. GANs excel in learning the distribution of minority classes, resulting in the creation of diverse and realistic synthetic samples, surpassing the interpolation of existing data. Unlike traditional augmentation methods, which may lead to overfitting due to the replication of existing samples, GANs produce samples that deviate from the majority class. This enhances the model’s ability to generalize effectively and accommodate new data instances [27]. GAN training uses iterative optimization. The generator and discriminator are alternately updated using gradient descent to minimize loss functions. This makes the generator and discriminator compete throughout training. The game theory-inspired minimax loss function is the most frequent GAN loss function. Eq (1) calculates mini-max loss for a GAN with generator ***G*** and discriminator ***D*** [28].

$$L_{GAN}(G.D)=E_{x∼p_{data}}(x)[logD(x)]+E_{z∼p_{z}}(z)[log(1-D(G(z)))]$$ (1)

Where***x*** represents real data samples drawn from the true data distribution ***pdata(x)***, ***z*** represents random noise (latent vector) drawn from a prior distribution ***pz(z***)(often a uniform or normal distribution),*G(z)* is the output of the generator given the noise z generating synthetic samples, and ***D(x)*** is the discriminator’s output, representing the probability that***x*** is representing.

The generator minimizes this loss, while the discriminator maximizes it. After training, the generator produces more realistic data that confuses the discriminator, while the discriminator becomes better at distinguishing real from fake data. Conditional GANs for synthetic data generation, also known as CT-GAN, is a synthetic tabular data generator that was developed to solve several problems that were present in the classic GAN. CT-GAN exceeds every method that has been developed to this day and is at least 87.5% more effective than Bayesian networks [29].

### Time series neural network

This study employs two distinct types of Time Series Neural Networks which are the Nonlinear Autoregressive Exogenous Neural Network and the Nonlinear Autoregressive Neural Network. Subsequent sections will delve into detailed discussions of these networks.

### 1) Nonlinear Autoregressive Exogenous Neural Network (NARX-NN)

This Network predicts how things change over time [30]. In this case, we use a method called NARX-NN, which is good at giving accurate guesses. Here’s what NARX-NN time series means in this context:

y(t)=h(x(t−1).x(t−2).…….x(t−k).y(t−k).y(t−1).y(t−2).…….y(t−p))+ϵ(t) (2)

The anticipated time series s*(t)*, is determined by the past value p and is influenced by an additional external time series, *x(t)*. The external time series *(t)*, might either have a single dimension or be multi-dimensional. The *NARX-NN* prediction model utilizes the previous output values along with exogenous input to estimate future values [31]. In this paper, the use of green finance is considered as the input time series at time *t*−1, denoted as *(t−1)*, while the nation variable is regarded as the exogenous input at time *t−1*, denoted as *x(t−1)*. The sole resultant is denoted as y(t). The *NARX-NN* and *NAR-NN* exhibit significant similarities. The country variable serves as an exogenous input in the *NARX* model.

### 2) Nonlinear Autoregressive Neural Network (NAR-NN)

Linear mathematical models struggle to capture the complexities of real-world economic scenarios, particularly in forecasting the growth of green finance. These complexities often involve numerous challenges and random fluctuations. To address this limitation, a nonlinear model, as represented by Eq (3), is necessary to predict the magnitude of these fluctuations in green finance growth. One such powerful tool for nonlinear time series forecasting is the Non-linear AutoRegressive Neural Network (NARNN) described in [32].

$$y(t)=f(y(t-1),\;y(t-2),\;y{\left(t-3\right)}_{\ldots },y(t-n))+\in (t)$$ (3)

In this case, *y* is the green finance data series at a time *t*, *n* is the green finance data series input delay, and *f* is a transfer function. The neural network is trained to learn the underlying function. This is achieved by adjusting the weights of connections between neurons and the biases of individual neurons to minimize the difference between the network’s predictions and the actual function’s outputs. The *y-* series of green finance was found by getting close to the term (t), ∈ which stands for “error tolerance.”

The following is a way to describe *NARNN’*s endogenous input.

$$\hat{y}(t)=f(y(t-1),\;y(t-2),\;y\left(t-3\right),\ldots y(t-20))+\in (t)$$ (4)

where delay of input n = 20. NAR-NN consists of one input layer, one or more hidden layer(s), and one output layer.

*NARNN* is recurrent and dynamic due to the connection of feedback. In this study, we used the narnet() built-in function for *NAR-NN* to implement the hyperbolic tangent (tansig, (5)) and sigmoid (logsig, (6)) functions to compare the network accuracies in the context of green finance forecasting.


$$O_{tansig}=\frac{e^{u}-e^{-u}}{e^{u}+e^{-u}}$$ (5)

$$O_{logsig}=\frac{1}{1+e^{-u}}$$ (6)

### The Augmented Dickey-Fuller test (ADF)

The Augmented Dickey-Fuller test (ADF) falls under the category of statistical tests known as unit root tests. Certain stochastic processes, like random walks, possess unit roots, which can complicate statistical inference when utilizing time series models. A unit root indicates non-stationarity and doesn’t always exhibit a trend [33]. The ADF test is an ‘augmented’ version of the Dickey Fuller test, it allows for higher-order autoregressive processes by including $\Delta \gamma_{t-p}$

$$y_{t}=c+\beta t+\alpha y_{t-1}+\emptyset_{1}\Delta \gamma_{t-1}+\emptyset_{2}\Delta \gamma_{t-2}\ldots \ldots +\emptyset_{p}\Delta \gamma_{t-p}+e_{t}$$ (7)

ADF tests yield statistics and p-values. At 1%, 5%, and 10% significance levels, the test statistic is compared to important values. Decide whether to reject the null hypothesis and declare the time series stationary if the test statistic is less than a predetermined number. As a result, you cannot rule out the null hypothesis, which suggests that there is a unit root in the time series if the test statistic is less negative than this crucial value. The p-value indicates the probability that a test statistic will be obtained that is equally or more extreme than the null hypothesis that was observed. Reject the null hypothesis and, if the p-value is less than the predetermined significance level, conclude that the time series exhibits stationarity. On the contrary, the null hypothesis cannot be rejected if the p-value surpasses the predetermined significance level; this would suggest the existence of a unit root in the time series [33].

## The proposed prediction model architecture

**Fig 1:**
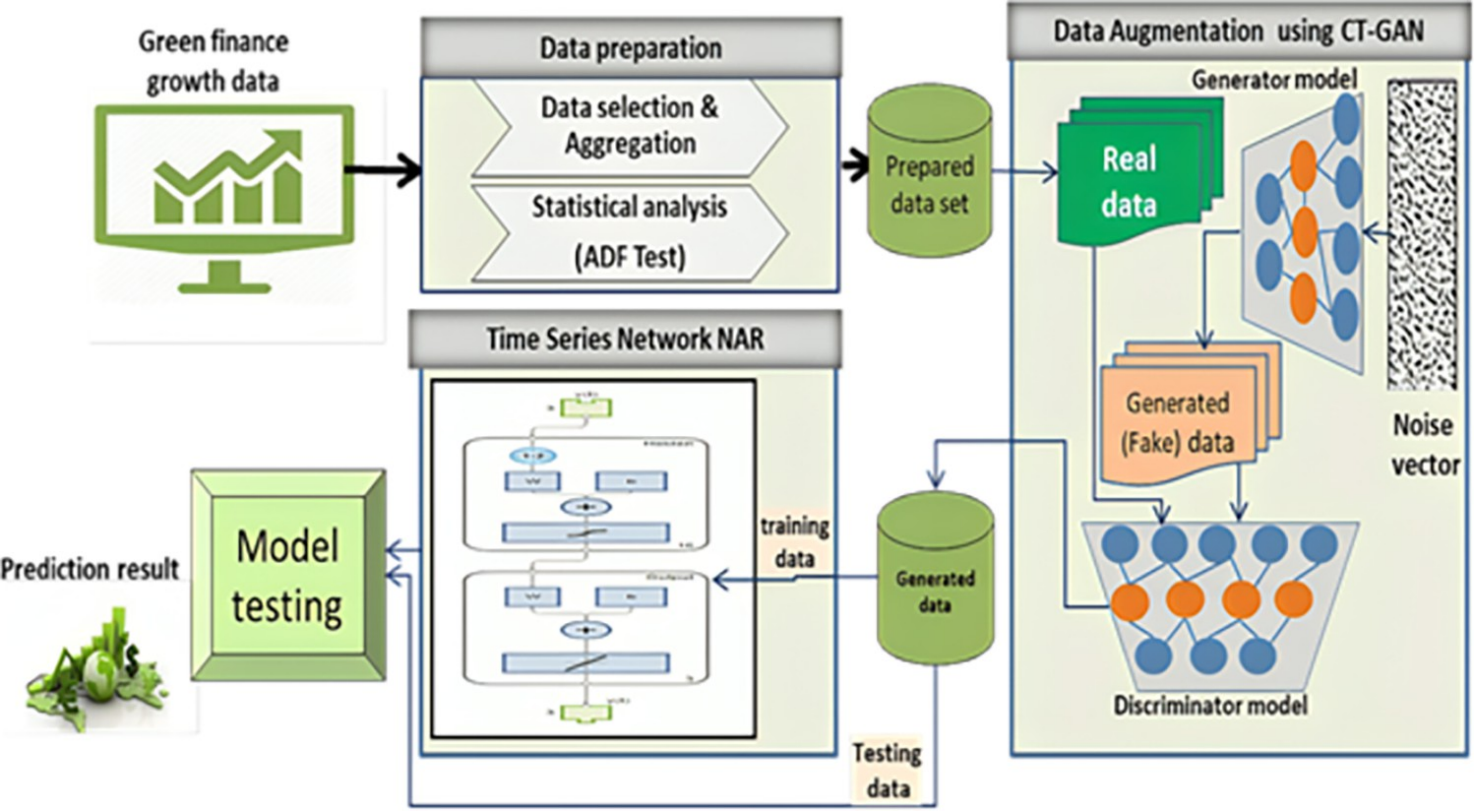
The general architecture of the proposed prediction model.

A generic preview of the proposed model architecture is presented in Fig 1. It consists of three main phases: data preparation, data augmentation using *CT-GAN*, and prediction phase using time series network NAR. Algorithm 1 presents the prediction model algorithm, and the next sections present these phases in detail.

**Algorithm 1: green finance prediction model**

**1. Read the dataset**.

**2. Data aggregation group by continent**

**3. Perform ADF test to select the appropriate prediction model**

**4. For each continent’s countries (3 continent)**

 • **Generate a fake data from real data using a generator and discriminator models that calculates minimax loss for a GAN with generator G and discriminator D**

$L_{GAN}(G.D)=E_{x∼p_{data}}(x)[logD(x)]+E_{z∼p_{z}}(z)[log(1-D(G(z)))]$

 • **Update the dataset** 

 • **Construct  NARNN using  narnet()** 

 • **Train the narnet to predict the green finance amount**. 

 • **Evaluation the performance based MSE, R2**

**5. Compare the performance with other time series approaches**

### Data preparation

This phase is crucial in readying the data for analysis. It involves two key processes: selecting and aggregating data and conducting statistical analyses to identify the most appropriate prediction model.

#### Data selection and aggregation

The studied data set includes data green finance data from 40 countries across 5 continents spanning several years, obtained from [34]. Table 2 provides a sample of the data for Denmark, a European nation. While the data included entries from various continents, Europe and Asia had the most comprehensive coverage. Preprocessing was necessary due to the presence of categorical data (shown in Table 3). Additionally, data for different countries were scattered throughout the dataset. To address this, we implemented a two-step organization process. The first step consists of identifying the continent for each country and grouped them into separate files. This analysis revealed that only Europe and Asia had sufficient data for further analysis. The second step is related to data transformation where categorical data is transformed into numerical values.

**Table 2:** Sample of the dataset.

year	Country	green finance
2013	Denmark	491.068993
2014	Denmark	287.234455
2015	Denmark	333.685167
2016	Denmark	324.362851
2017	Denmark	300.12899
2018	Denmark	287.217543

**Table 3:** Categories of the dataset.

Europe		Asia		Various countries	
# courtiers	Dataset size	# courtiers	Dataset size	# courtiers	Dataset size
24	249	9	102	7	66

**Fig 2:**
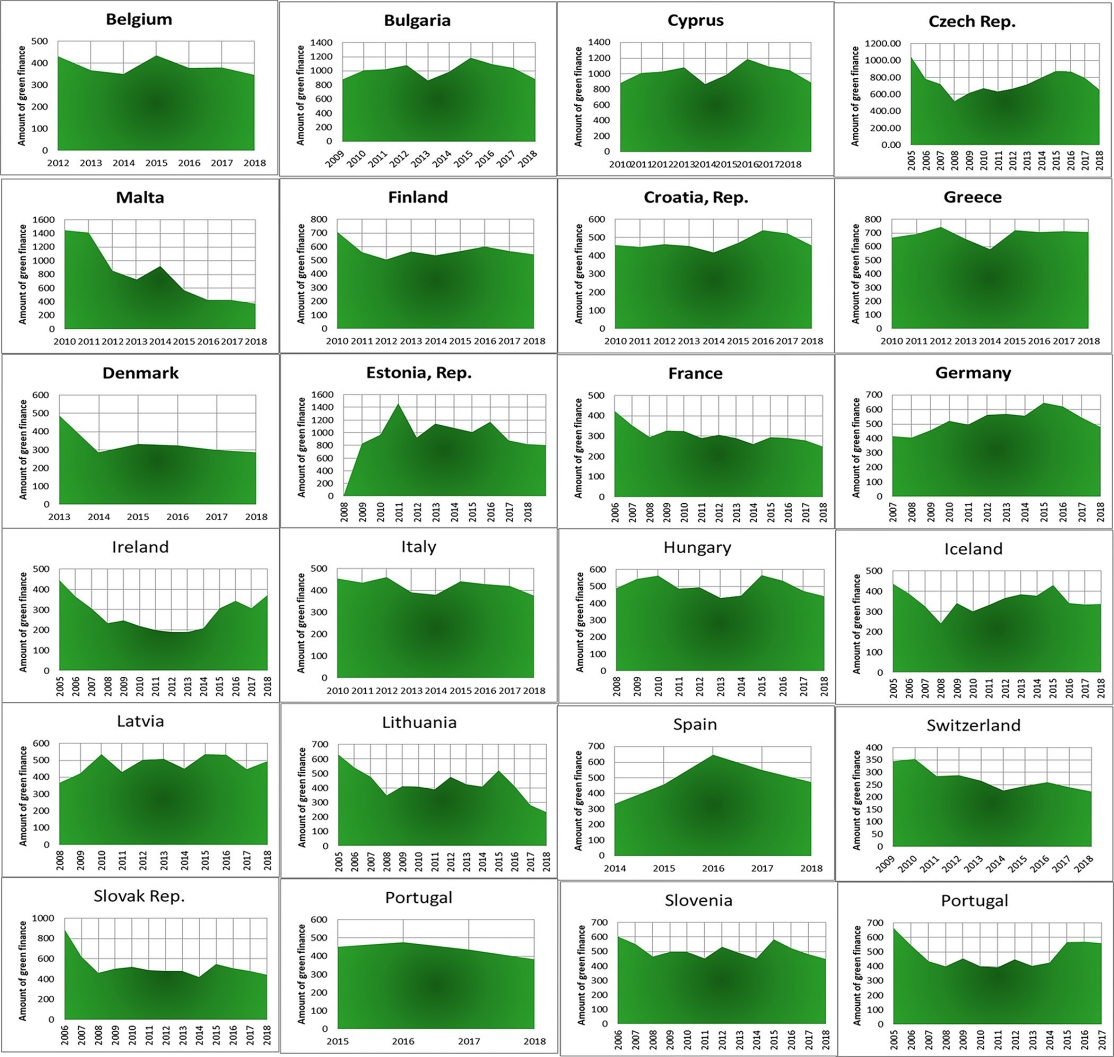
European green finance growth.

**Fig 3:**
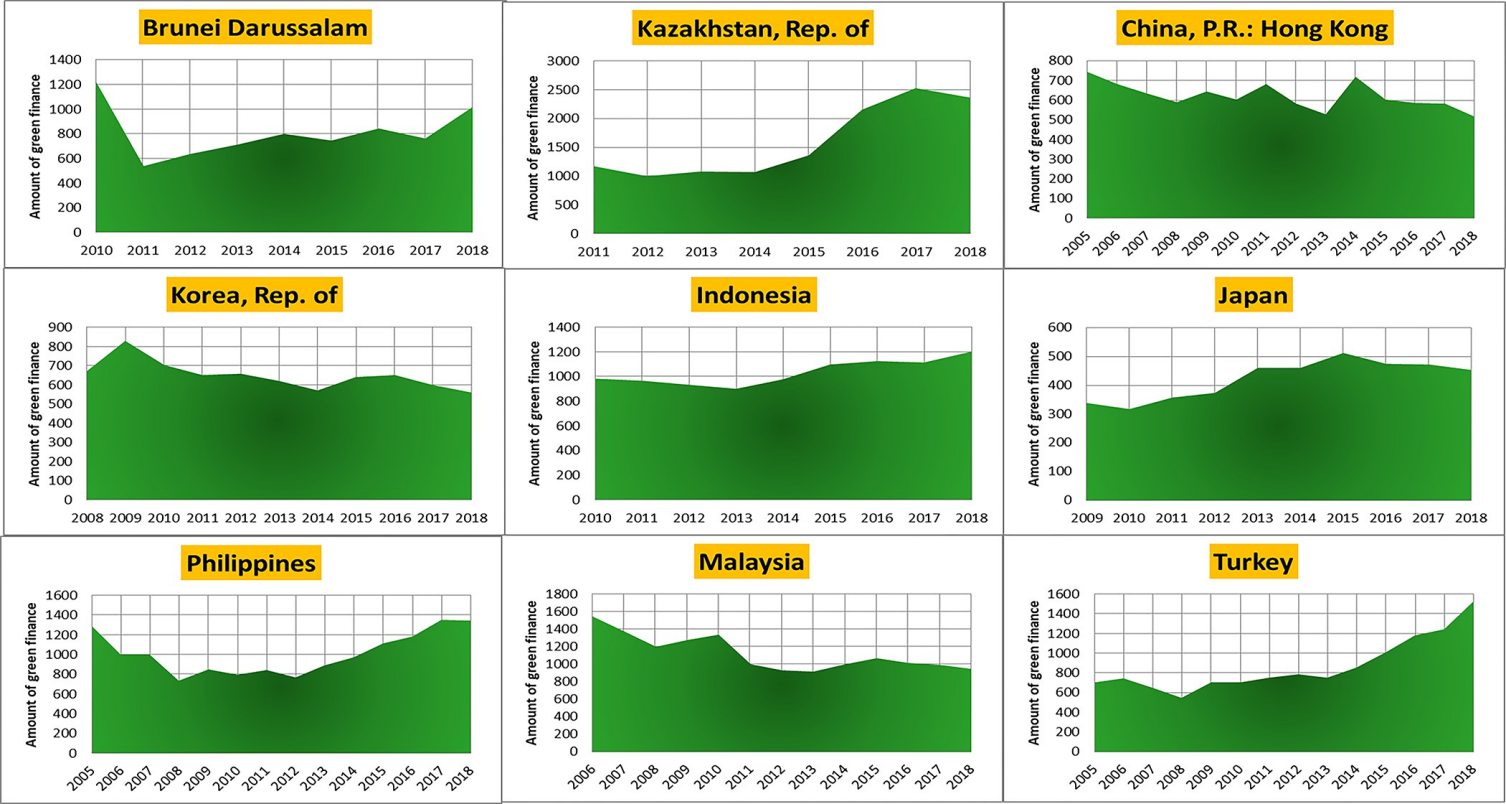
Asian green finance growth.

**Fig 4:**
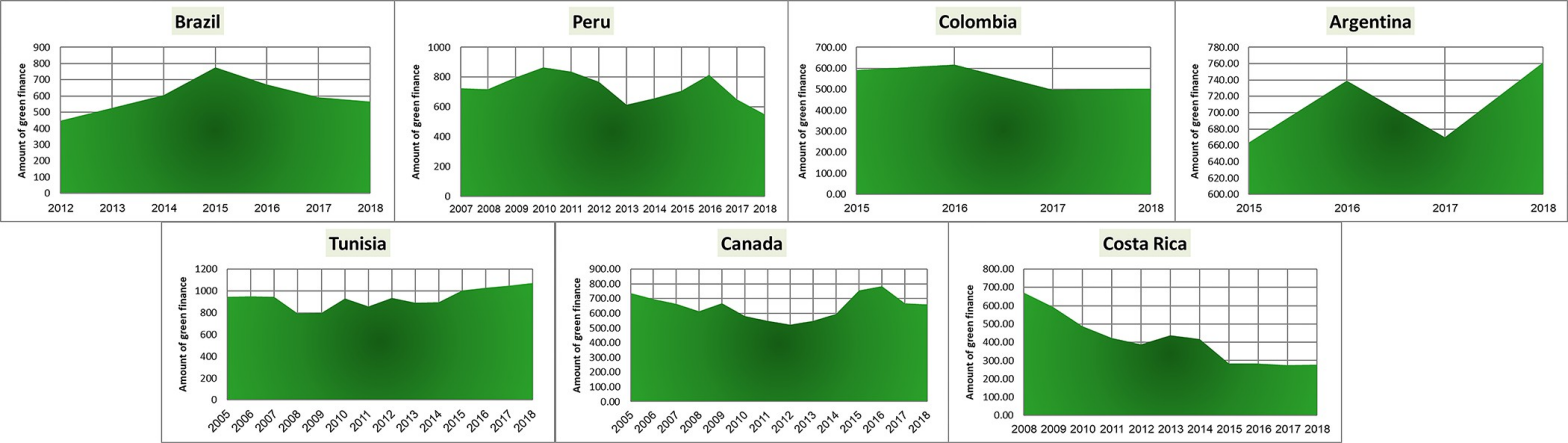
Various countries’ green finance growth.

Preliminary experiments indicated the need for data augmentation. Consequently, we employed CT-GAN (likely referring to Conditional Generative Adversarial Network) to augment the dataset as the final preprocessing step. The visualizations of green finance growth in Europe and Asia are presented in Figs 2–4.

#### Statistical analysis

Time-series data is valuable for analysis and prediction because it captures trends and patterns that change over time. However, stationary data, which exhibits little change over time, lacks these patterns and isn’t ideal for forecasting. Therefore, it’s crucial to assess data stationarity before proceeding.

**Fig 5:**
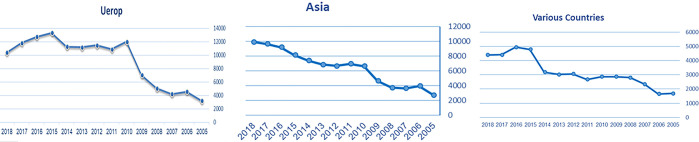
(a) Growth in green finance in Europe (b) Growth in green finance in Asia (c) Growth in green finance in Various Countries.

To analyze stationarity in our green finance growth data for Europe, Asia, and various countries, we first visualized it. Fig 5(A)–5(C) display the plots for each region, respectively. These visualizations suggest that the data might be non-stationary. To confirm our suspicions, we will employ the Augmented Dickey-Fuller statistical test, a robust method for detecting stationarity [33]

The Augmented Dickey-Fuller (ADF) test results, presented in Table 4, reveal that the green finance growth data across all regions (Europe, Asia, and Other Countries) exhibits non-stationary characteristics. This implies that the data lacks consistent trends or patterns over time.

**Table 4:** The results of the augmented dickey-fuller test.

Europe	’Test Statistic ’	-1.797584
	’p-value’	0.381662
	’Critical Value (1%)’	-4.068854
	’Critical Value (5%)’	-3.127149
	’Critical Value (10%)’	-2.701730
Asia	’Test Statistic’	0.310327
	’p-value’	0.977813
	’Critical Value (1%) ’	-4.665186
	’Critical Value (5%)’	-3.367187
	’Critical Value (10%)’	-2.802961
various countries	’Test Statistic’	-1.111513
	’p-value’	0.710459
	’Critical Value (1%)’	-4.068854
	’Critical Value (5%)’	-3.127149
	’Critical Value (10%)’	-2.701730

For each category, the test statistic is higher than the critical values at various significance levels, and the corresponding p-values all exceed the chosen significance level of 0.05. In statistical terms, these results fail to reject the null hypothesis of non-stationarity. Consequently, the green finance growth data cannot be directly used for traditional forecasting methods that rely on stationary data.

Hence, to effectively predict future green finance growth patterns, this study proposes using a Nonlinear AutoRegressive Neural Network (NARNN) model. This type of model is well-suited for analyzing and predicting non-stationary time-series data.

## Experimental results and analysis

To optimize the network’s performance, we employed an iterative approach, evaluating different configurations through multiple tests. The most accurate results were achieved with a single hidden layer containing 20 neurons. We opted for the Levenberg-Marquardt Backpropagation (LMBP) algorithm for training due to its efficiency [35].

Since our goal was one-step-ahead forecasting, a simpler architecture was chosen compared to the typical closed-loop structure used for multi-step predictions. The effectiveness of the final three network configurations was assessed using Mean Squared Error (MSE), Root Mean Squared Error (RMSE), and R-Squared (R^2^).

MSE is a common metric in regression tasks. It measures the average squared difference between predicted values and actual targets (Eq 8). It’s important to note that MSE tends to inflate the impact of small errors due to the squaring, potentially overstating the model’s shortcomings [11].

$$MSE=(\frac{1}{N})\sum_{i=1}^{N}{\left({\hat{y}}_{i}-y_{i}\right)}^{2}$$ (8)

To assess the prediction accuracy, *N* represents the total number of test samples, where y_i_ denotes the *i*th test sample, and $\hat{y}$ stands for the predicted value of y_i_. MSE serves as an indicator of the precision of the forecasting results, with a smaller MSE indicating a more accurate forecast.

As shown in Eq 9, the Root Mean Squared Error (RMSE) is utilized to compute the discrepancy between the actual and observed values.

$$RMSE=\sqrt{(\frac{1}{N})\sum_{i=1}^{N}{\left({\hat{y}}_{i}-y_{i}\right)}^{2}}$$ (9)

Where *N* is the number of test samples that subscribe to the i^th^ test sample, and ${\hat{y}}_{i}$ is the predicted value of $y_{i}$.

Because RMSE uses the average error, it is susceptible to aberrant points. The RMSE value is greatly affected if the regression value of a point is not credible, since this will result in a relatively large error. The more accurate the predicted results, the smaller the RMSE. Moving on to R-square (R2), its primary objective is to measure the degree of correlation between predicted and observed data. Consider a dataset comprising *n* values labeled y_d2_, y_d3_, …, y_n_ (often denoted as y_i_ or represented as a vector y = (y1, y2,…, y*n*) ^T^, each corresponding to a predicted value f_1_, f_2_,…, f_n_. To compute both the total sum of squares and the sum of squares remaining, employ Eq (10) and Eq (11) as follows:

The total of all squares:

$$S_{t}=\sum_{i=1}^{n}{\left(y_{i}-{\hat{y}}_{i}\right)}^{2}$$ (10)

The sum of residual squares is another name occasionally used to refer to the sum of squares. The definition of it is as follows.

$$S_{re}=\sum_{i=1}^{n}{\left(y_{i}-f_{i}\right)}^{2}$$ (11)

The most common expression of the coefficient of determination is given in Eq (12).

$$R^{2}=1-\frac{S_{t}}{S_{re}}$$ (12)

The low values of MSE are indicative of optimal performance. On the other hand, a high R2 signified a considerable degree of accuracy [11]. All experiments will be listed as follows: The low values of MSE indicate the best result. In contrast, the high value of R^2^ indicated a high accuracy.

This paper presents four implemented experiments. They are as follows:

- Experiment (1) Data augmentation using CT-GAN
- Experiment (2) Prediction using NARX- NN
- Experiment (3) Predicting the green finance using NAR- NN for the original data.
- Experiment (4) Predicting the green finance using NAR- NN for the original data.

### Experiment implementation

Experiments are presented in the following sub-sections; the first experiment is carried out for data preparation, and the remaining experiments are carried out to obtain the most accurate findings possible for the prediction process. All these experiments are discussed as follows.

### Experiment (1): Data augmentation using *CT-GAN*

The experiments were conducted using TensorFlow and Keras on a TPU through Google Colab. To address the issue of limited data, this experiment was initiated. The ***CT-GAN*** architecture employs a conditional generator to produce rows conditioned on a single discrete column. It samples training data based on the log-frequency of each category, rather than randomly sampling, thereby ensuring a more balanced representation of minor categories within highly imbalanced categorical columns. This approach aids the GAN model in exploring discrete values evenly.

**Fig 6:**
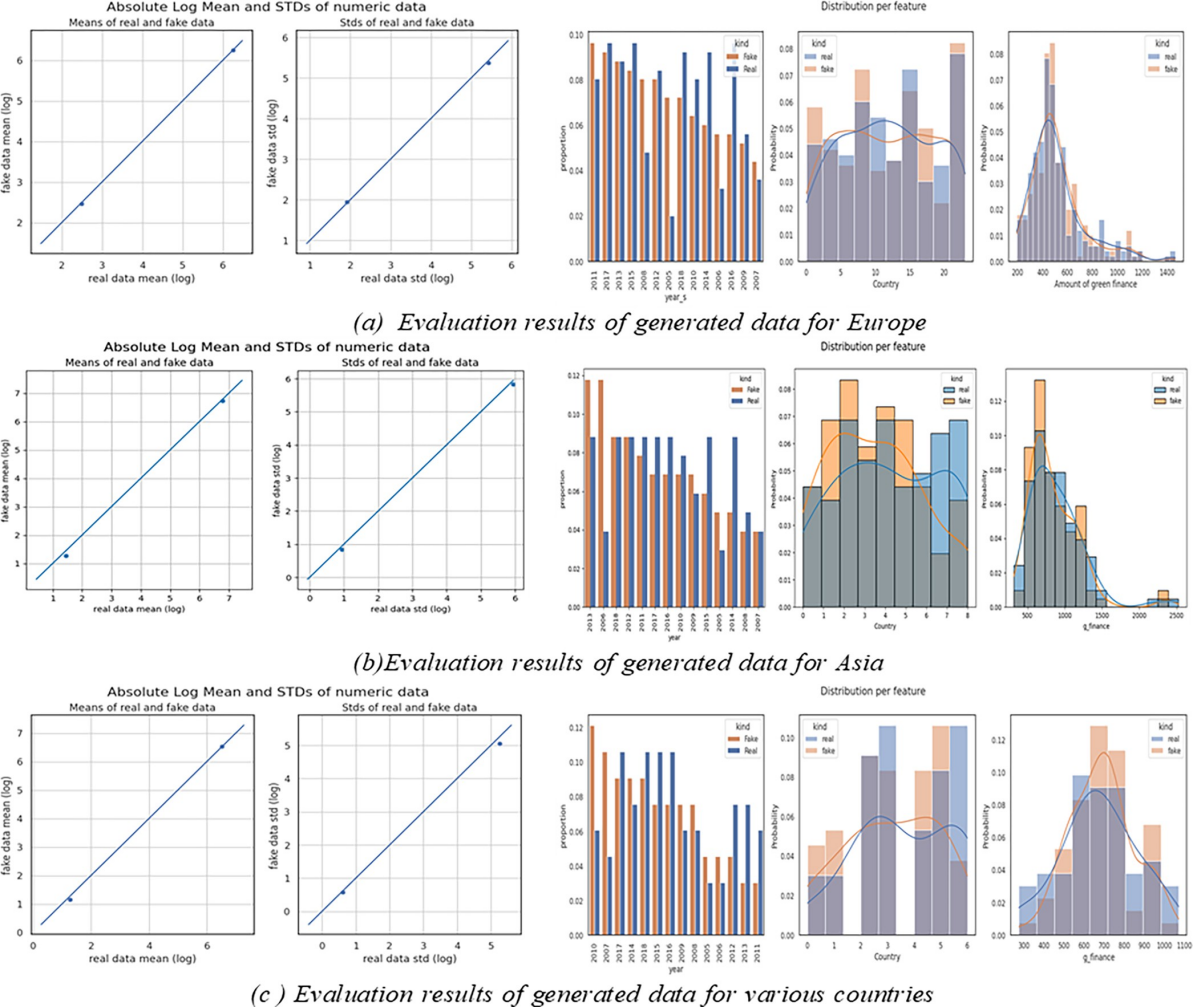
Experiment (1): Data augmentation using CT-GAN.

Additionally, for tabular data, unlike pixel data in images, continuous variables may exhibit non-Gaussian or complex multi-modal distributions. CT-GAN addresses this by normalizing continuous columns based on their mode. In this experiment, 1000 data points were generated for Europe, Asia, and various distributed countries. To evaluate the generated fake data and its similarity to the read data, it shows the evaluation results for European countries in Fig 6(A), Asian countries in Fig 6(B), and other various countries in Fig 6(C), they all show the clear similarities between real and fake generated data.

### Experiment (2): Prediction using NARX- NN

**Fig 7:**
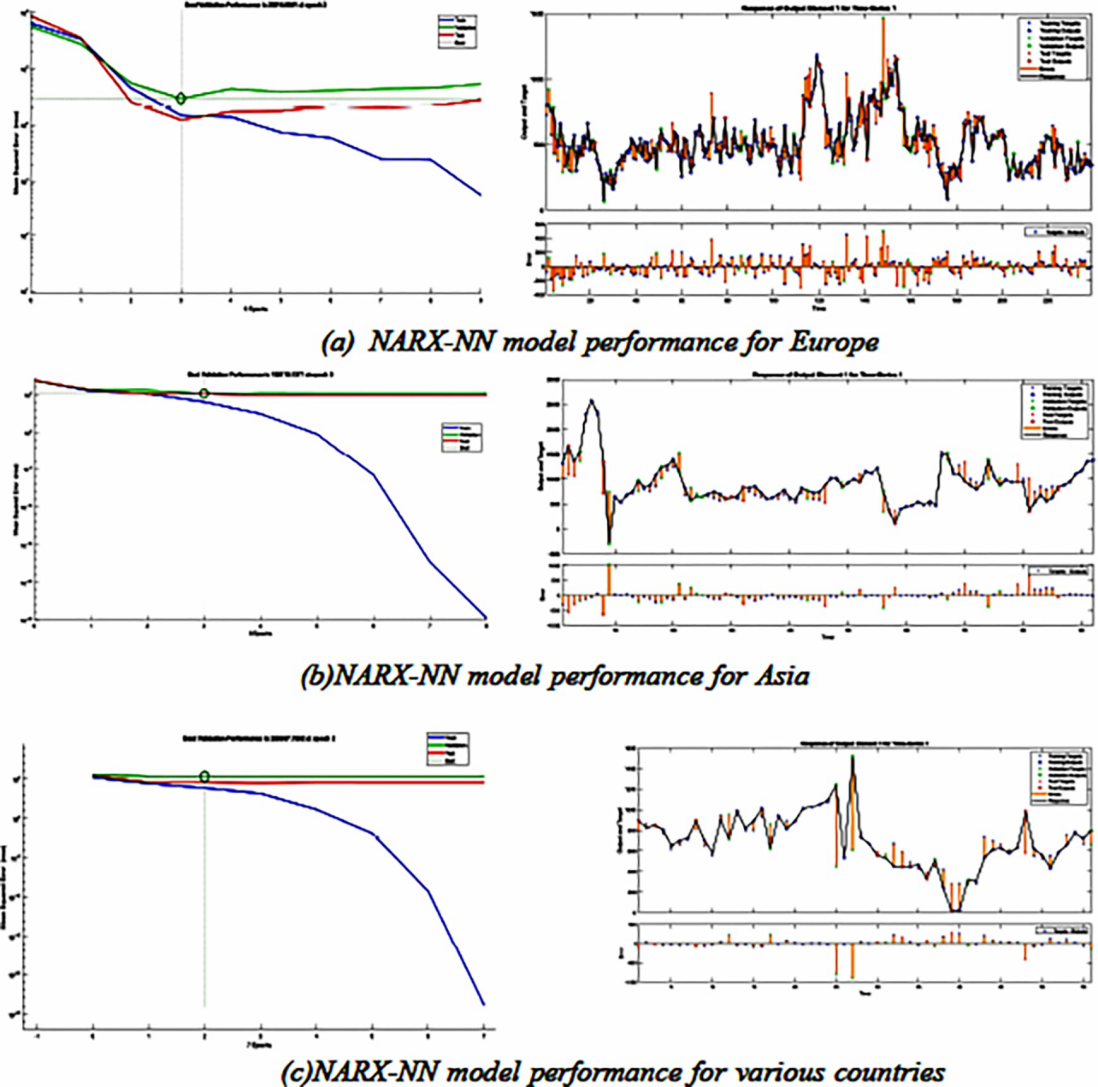
Experiment (2) Prediction using *NARX-NN*.

The experiment and subsequent ones were conducted using MATLAB (version R2023a) as the implementation platform. In this experiment, NARX-NN was employed to forecast the effects of carbon dioxide emissions and pollution on investment and capital flows. Once the data preparation phase was completed, the primary dataset was used for this experiment. Predictions were made for various countries, encompassing those from Europe, Asia, and other regions. Fig 7 depicts the performance of the model for each of the three categories. This experiment’s R^2^ test yielded findings of 74%, 52%, and 86%, respectively, for Europe, Asia, and other regions.

### Experiment (3): Predicting the green finance using *NAR- NN* for the original data

**Fig 8:**
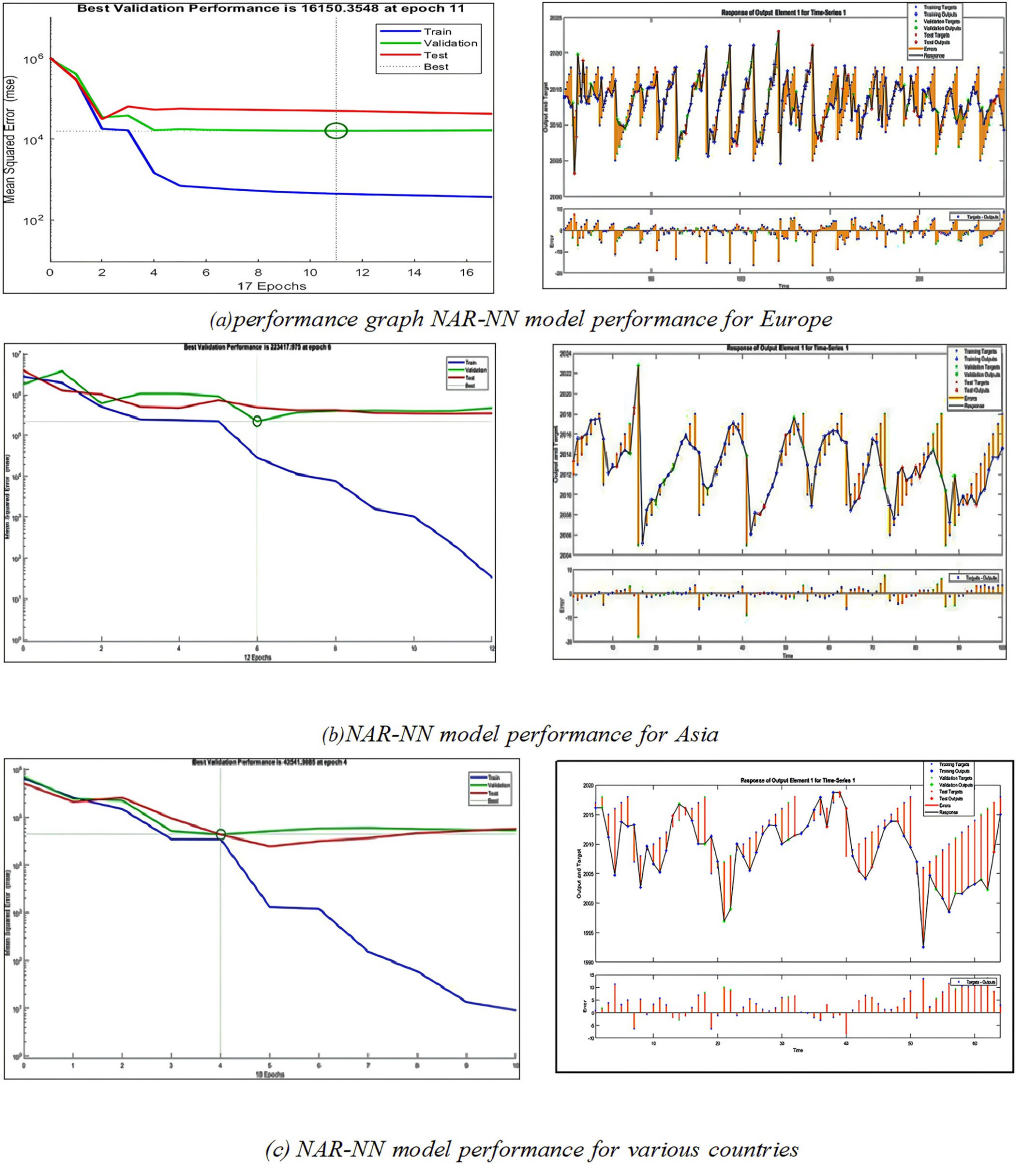
Experiment (3): prediction using *NAR-NN* without data augmentation.

As a result of the disappointing outcome of the prior experiment, it has been determined to make use of the NAR-NN model because it will be appropriate for the characteristics of the data. NAR-NN was utilized to make predictions on the impact that carbon dioxide emissions and pollution have on investment and capital flows. The experiment was carried out on the primary dataset once the preparation has been completed without data augmentation. The experiment on making predictions was carried out for several countries, including those in Europe, Asia, and other regions. Fig 8 depicts the performance of the model for each of the three categories. The R^2^ test was performed in this experiment, and the findings showed that it was acceptable; the results were 96%, 73%, and 97%, respectively, for Europe, Asia, and other regions.

### Experiment (4) Predicting the green finance using *NAR- NN* for the original data

**Fig 9:**
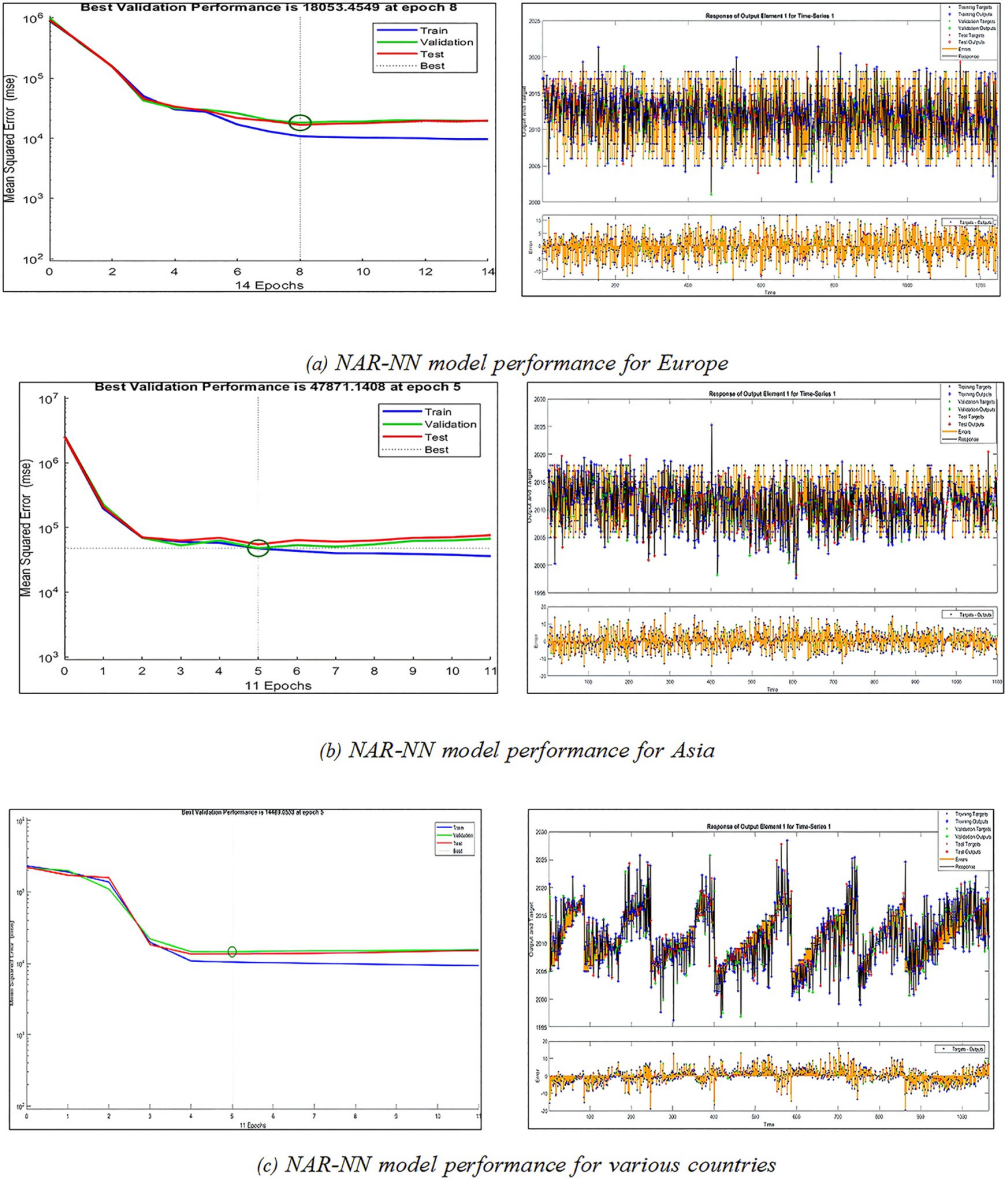
Experiment (4): Prediction using *NAR-NN* based *CT-GAN* data augmentation.

The results of the previous experiment were acceptable. However, they were not satisfactory enough; this could be due to the limited amount of data that was trained on. In this experiment, we use data augmented with CT-GAN, and the *NAR-NN* model will be applied for prediction. The experiment on making predictions was conducted out for a variety of countries, including those in Europe, Asia, and other regions of the world. The performance of the model is depicted in Fig 9 for each of the three different categories. The R^2^ test was carried out, and the results were successful with the values 98.8%, 96.6%, and 99% for Europe, Asia, and other regions respectively.

### Results analysis

The findings of all of the experiments are presented in Table 5. While the training R^2^ result for the *NAR-NN* model without data augmentation is superior to the R^2^ result of the *NARX-NN* model without data augmentation, the training results after the data augmentation are lower than the *NAR-NN* model without data augmentation. This is the case for the model used in European countries. On the other hand, the test and validation R^2^ results for the *NAR-NN* model with *CT-GAN* data augmentation yield the greatest results 98.7% and 98.8% respectively.

**Table 5:** The results the of the all experiments.

		NARX-NN model without data augmentation			NAR-NN model without data augmentation			NAR-NN model with CT-GAN data augmentation		
		MSE	RMSE	R^2^%	MSE	RMSE	R^2^%	MSE	RMSE	R^2^%
**Europe**	training	1.48E+04	1.22E+02	86	445.2	2.11E+01	99.9	1.07E+04	1.03E+02	99.2
	validation	2.92E+04	1.71E+02	71.3	1.60E+04	1.26E+02	98	1.80E+04	1.34E+02	98.7
	test	1.23E+04	1.11E+02	74	5.02E+04	2.24E+02	96	1.60E+04	1.26E+02	98.8
**Asian**	training	1.05E+04	1.02E+02	97	2.87E+04	1.69E+02	98	4.80E+04	2.19E+02	96.6
	validation	1.35E+05	3.67E+02	61	2.23E+05	4.72E+02	87.6	4.89E+04	2.21E+02	96.5
	test	1.32E+05	3.63E+02	52	4.90E+05	7.00E+02	73	4.66E+04	2.16E+02	96.6
**various countries**	training	6.80E+03	8.25E+01	98	3.46E+04	1.86E+02	97.7	1.03E+04	1.01E+02	99.2
	validation	2.05E+05	4.53E+02	11	4.30E+04	2.07E+02	96.8	1.44E+04	1.20E+02	98.9
	test	3.43E+04	1.85E+02	86	4.30E+04	2.07E+02	97.2	1.35E+04	1.16E+02	99

The model used for the Asian countries is comparable to the approach used with the European countries. The R^2^ value of the NAR-NN model without data augmentation is higher than the R^2^ value of the *NARX-NN* model without data augmentation. Nevertheless, the implementation of data augmentation has led to reduced training results for the *NAR-NN* model compared to when data augmentation was not used. However, the *NAR-NN* model, when combined with *CT-GAN* data augmentation, achieves the highest R^2^ result during both the test and validation phases.

Regarding R^2^ final results for training, validation, and testing, the *NAR-NN* model augmented with CT-GAN data yields the highest values for the models of different countries are 99.2%, 98.9%, and 99%, respectively.

Based on the analysis of the results, the *NAR-NN* model outperforms other models in all three continents. This is consistent with our previous statistical analysis, which recommended that the *NAR-NN* model is the most suitable for prediction. The proposed *CT-GAN* also had a significant positive impact on enhancing the results.

The proposed model enhances market confidence by providing reliable forecasts of green finance growth, reducing uncertainty, and attracting more investment. This research contributes to economic resilience by diversifying economic portfolios, creating new job opportunities, and stimulating technological advancements. The insights can inform evidence-based policies to accelerate the transition to a sustainable economy, such as targeted incentives and subsidies. Green finance also yields societal benefits, such as mitigating environmental degradation and improving public health. By aligning financial interests with environmental objectives, this research contributes to sustainable development and a prosperous future.

## Conclusion and future work

Climate change, driven by rising atmospheric carbon dioxide levels, poses a significant environmental threat. In response, environmentally responsible finance, or "green finance," has emerged as a critical tool. While research on weather and stock prices remains limited, the link between air pollution and financial markets is gaining recognition. Machine learning techniques, particularly time series neural networks, offer more promising forecasting abilities compared to traditional models in financial analysis.

This study aims to predict the future trajectory of green finance and encourage investments in green projects. Notably, the relationship between pollution levels and green finance investments has not been extensively explored. To the best of our knowledge, the relationship between pollution and investments in green finance, particularly, has not been addressed in the literature.

Our research leverages neural time series methods to assess green finance effectiveness across different periods. To address the challenges of analyzing and forecasting investment data from various continents over time, we employed a Nonlinear AutoRegressive Neural Network (NARNN) and similar techniques. We utilized machine learning to predict green finance investments in key continents–Asia and Europe. The data was augmented using a Generative Adversarial Network (GAN) before applying neural time series prediction. The achieved R-squared (R^2^) values of 99% for both Europe and Asia demonstrate the feasibility and accuracy of our proposed approach.

Future work can involve expanding the study by collecting more data from additional continents like Africa and America. Additionally, incorporating more country-specific features can provide deeper insights into factors influencing green finance.
